# Tobacco control in low-income and middle-income countries: findings from WHO FCTC investment cases

**DOI:** 10.1136/tc-2024-058717

**Published:** 2024-05-02

**Authors:** John A Tauras

**Affiliations:** Department of Economics, University of Illinois Chicago, Chicago, Illinois, USA

**Keywords:** Economics, Low/Middle income country, Public policy

The health consequences of tobacco consumption have been the subject of rigorous scientific research for over 70 years. Despite the overwhelming evidence that has accumulated on the deleterious health effects of tobacco smoking, over 1.1 billion people worldwide smoked tobacco regularly in 2020.^1^ Current tobacco consumption is concentrated in low-income and middle-income countries (LMICs), with approximately 80% of people who use tobacco now living in LMICs.^2^ Indeed, tobacco use is one of the leading causes of preventable death and disease worldwide. Each year, tobacco claims the lives of over 8 million people, including an estimated 1.3 million people who do not smoke but are exposed to secondhand smoke.^3^

Efforts to decrease tobacco consumption in some countries date back to the 1960s following the first research published on the adverse health effects of cigarette smoking. However, the adoption of the WHO Framework Convention on Tobacco Control (FCTC) served as a catalyst for significant progress in global tobacco control. The FCTC was developed in response to the worldwide expansion of the tobacco epidemic. It was adopted by the World Health Assembly in 2003 and entered into force in February 2005. The FCTC is one of the United Nations treaties with the broadest adoption worldwide. Currently, there are 183 Parties to the FCTC, covering more than 90% of the world population.^4^

The FCTC presents a comprehensive plan for member nations to tackle the tobacco epidemic and provides a diverse range of evidence-based measures aimed at reducing both the demand for tobacco (Articles 6–14) and the supply of tobacco (Articles 15–17).^5^ The core demand reduction strategies^5^ consist of price and tax measures to reduce the demand for tobacco, as well as non-price measures, including the following:

- Protection from exposure to tobacco smoke.
- Regulation of the contents of tobacco products.
- Regulation of tobacco product disclosures.
- Packaging and labelling of tobacco products.
- Education, communication, training and public awareness.
- Tobacco advertising, promotion and sponsorship.
- Demand reduction measures concerning tobacco dependence and cessation.

The core supply reduction strategies^5^ include measures related to the following:

- Illicit trade in tobacco products.
- Sales to and by minors.
- Provision of support for economically viable alternative activities.

Despite being signatories to the WHO FCTC, numerous recommended policies and articles remain unimplemented in many nations.^6^ Additionally, even when these measures are enacted into law, they often suffer from inadequate enforcement.^6^

The FCTC 2030 programme is an effort to expedite the implementation of WHO FCTC articles across 15 LMICs in phase 1 and an additional 9 LMICs in phase 2. The FCTC 2030 programme aims to fortify tobacco control governance primarily by implementing FCTC Article 6, which involves increasing tobacco taxation; implement the two FCTC measures on tobacco packaging (Article 11) and on ending tobacco advertising, promotion and sponsorship (Article 13); and support the implementation of other WHO FCTC articles that are deemed national priorities.^7^ Moreover, the FCTC 2030 programme aims to bolster tobacco control efforts, enhance capacity, garner support for stronger tobacco control legislation and improve implementation of the national tobacco control plans in phase 1 countries.^7^ As part of their applications to FCTC 2030, countries requested a range of support from the project’s priority areas. Each country participating in FCTC 2030, however, requested a tobacco control investment case.^8^ Investment cases attempt to quantify both the direct and indirect benefits of implementing tobacco control interventions and then evaluate these benefits against the costs of implementing the interventions. The investment cases provide countries with essential data regarding the costs of tobacco use and the net benefits of implementing tobacco control measures, and may provide a politically feasible route for bolstering the implementation of WHO FCTC measures when the net benefits of investments in tobacco control are positive.

The research featured in this supplement details 21 national tobacco investment cases carried out between 2017 and 2022 as part of the FCTC 2030 initiative. Small *et al* 8 describe the tobacco control advancements that occurred during and following the tobacco investment cases in the following LMICs: Armenia, Cabo Verde, Cambodia, Chad, Colombia, Costa Rica, El Salvador, Eswatini, Georgia, Ghana, Jordan, Laos, Madagascar, Myanmar, Nepal, Samoa, Sierra Leone, Sri Lanka, Suriname, Tunisia and Zambia. Small *et al* 8 found that tobacco control advancements were observed in 16 of the 21 LMICs where the investment cases were carried out. The positive net benefits identified in the investment cases likely contributed to the tobacco control advancements that were observed in the 16 LMICs.

Previous studies aiming to quantify the economic effects of tobacco consumption have employed varied methodologies, leading to challenges in comparing results across different studies. Nugent *et al* 9 describe a common investment case methodology that was used to measure the economic impacts of tobacco usage and the return on investment (ROI) stemming from tobacco control efforts in 36 countries from 2017 to 2022. The economic analysis used in Nugent *et al* 9 initially calculated a 1-year tobacco attributable socioeconomic loss in each of the 36 countries and then assessed the extent to which tobacco control measures could reduce the burden of tobacco. The ROI analysis compared two scenarios: a status quo scenario where the 1-year losses are extended year-over-year with no changes in tobacco control, and an interventional scenario showing the results that could be achieved by implementing tobacco control measures. The common investment case methodology used in Nugent *et al* 9 enabled cross-country comparisons of the results and unveiled both obstacles and avenues for enhancing future tobacco control efforts.

Mann *et al* 10 provide a summary of results and a synthesis of findings from 21 tobacco investment cases in LMICs that employed the common investment case methodology described in Nugent *et al*.^9^ Tobacco consumption was found to result in average annual losses of US$95 million, US$610 million and US$1.6 billion in the low-income, lower-middle-income and upper-middle-income countries that were studied, respectively. These losses represent 1.1%, 1.8% and 2.9% of the average annual national gross domestic product of low-income, lower-middle-income and upper-middle-income countries, respectively. In all 21 investment cases, the ROI for the demand reduction policy package was found to be greater than one, suggesting the benefits of the tobacco control efforts significantly outweighed the costs involved in implementing tobacco control measures. Among all the tobacco policy interventions, increasing taxes of tobacco products was consistently found to have the highest ROI. This is not surprising given the effectiveness of taxes in reducing smoking prevalence rates and the relatively affordable implementation costs.

Spencer *et al* 11 present findings from investment case equity analyses across 19 LMICs. Using smoking prevalence data by income quintiles, Spencer *et al* 11 estimated the effects of a 30% increase in the most sold brand of cigarettes on smoking prevalence, mortality and spending on cigarettes. In all countries but one, a 30% increase in the price of cigarettes led to the largest decline in smoking prevalence among the poorest 20% of the population. Moreover, across the 19 LMICs, 33% of the price-induced lives saved occurred in the poorest 20% of the populations. Given the price inelastic demand for cigarettes, the higher prices resulted in all income groups spending more money on cigarettes. However, the poorest 20% of people who smoke were found to pay an average of only 12% of the expenditure increase. The analysis by Spencer *et al* 11 demonstrated that increasing cigarette prices through taxation is progressive and disproportionately benefited the poor in the LMICs that were studied.

The research presented in this supplement is important. For many of the countries, the FCTC 2030 tobacco investment cases that were conducted as part of this supplement contain the first-ever national research on the economics of tobacco. In each of the investment cases that were conducted, a common methodology was employed. The benefits of investing in tobacco control were found to far outweigh the costs of implementation. The findings from this supplement provide policy makers, public health professionals and advocates needed insights into the health and economic benefits of implementing WHO FCTC policies and articles, and may help advance future tobacco control efforts in FCTC 2030 countries and other LMICs. Finally, as described above, tobacco taxation was found to have the highest ROI among all the tobacco control policies examined. Furthermore, increasing cigarette prices through taxation was found to disproportionately benefit the poor in the LMICs that were studied. While higher taxes on tobacco products are vital to tobacco control efforts, countries should not neglect other tobacco control policies with lower ROIs. Tobacco control policies tend to complement one another, working synergistically to reduce tobacco use. The impact of comprehensive tobacco control endeavours will undoubtedly result in a greater reduction in tobacco consumption compared with the implementation of a single policy alone.
